# Glypican-3 and Cytokeratin-19 Expression in Pancreatic Cancer in a Canadian Population

**DOI:** 10.3390/jcm13226893

**Published:** 2024-11-16

**Authors:** Carley Bekkers, Ravi Ramjeesingh, Thomas Arnason

**Affiliations:** 1 Division of Anatomical Pathology, Queen Elizabeth II Health Sciences Centre, Halifax, NS B3H 1V8, Canada; carley.bekkers@nshealth.ca; 2 Division of Medical Oncology, Queen Elizabeth II Health Sciences Centre, Halifax, NS B3H 3A7, Canada; ravi.ramjeesingh@nshealth.ca

**Keywords:** pancreatic neuroendocrine tumor, pancreatic adenocarcinoma, glypican-3, GPC3, cytokeratin-19, CK19

## Abstract

**Background/Objectives:** One study of pancreatic ductal adenocarcinoma has found expression of glypican-3 (GPC3) and cytokeratin-19 (CK19) determined by immunohistochemistry to be associated with higher stage and grade disease, with a more adverse prognosis. The reported 44% rate of GPC3 expression in pancreatic cancer raises the important possibility that targeted immunotherapies currently in development for hepatocellular carcinoma may also prove useful for GPC3-expressing pancreatic cancers. The present study aims to determine if a similar expression pattern of these markers and stage/grade/prognostic associations is present in our Canadian patient population. **Methods:** Patients with a pancreatic surgical resection for adenocarcinoma or neuroendocrine tumor (NET) were identified from pathology records over a 5-year period. Immunohistochemistry for GPC3 and CK19 was performed on archived tumor tissue and the proportion of positive cells and intensity of staining were recorded. Grade, stage, and overall survival were compared in patients with NETs that were CK19-positive versus -negative. **Results:** All 72 pancreatic adenocarcinomas and 20 NETs tested were negative for GPC3, apart from a single case of pancreatic adenocarcinoma. All 72 adenocarcinomas were positive for CK19 expression. Half of the NETs were positive for CK19. There was no correlation between CK19 expression in NETs and tumor grade, lymph node metastasis, distant metastasis, or overall survival. **Conclusions:** We are skeptical of the reported prognostic value of GPC3 and CK19 in pancreatic adenocarcinomas. CK19 as a prognostic marker in NETs has potential for further study. The results with our protocol for GPC3 immunohistochemistry suggest that pancreatic cancer may be a less promising target for GPC3-targeted immunotherapies than previously thought.

## 1. Introduction

Pancreatic ductal adenocarcinoma has one of the highest mortality rates of all human cancers. Survival is largely dependent on the possibility of surgical resection. However, the 5-year survival rate for patients who undergo surgical resection is estimated to only be 11–25% [1]. Although survival rates can be slightly improved with chemotherapy, side effects can be debilitating, outcomes remain poor, and options for systemic therapies beyond chemotherapy remain limited. Consequently, there is significant interest in identifying new therapeutic targets for immune-based systemic therapy in pancreatic cancer.

A 2016 study by Yao et al. raised the possibility of a new prognostic marker and, more importantly, a potential therapeutic target in pancreatic cancer—glypican-3 (GPC3) [2]. GPC3 is a cell-surface proteoglycan that is encoded by the GPC3 gene. It is involved in the regulation of cell growth and differentiation. While some cancer types exhibit downregulation of GPC3 expression, others, most particularly hepatocellular carcinoma (HCC), show an overexpression of GPC3. GPC3 immunohistochemistry has become recognized as a clinically helpful diagnostic marker for pathologists to distinguish hepatocellular carcinoma from benign hepatocellular tumors and from many non-hepatocellular neoplasms [3].

The Yao et al. [2] research group found a high (44%) rate of expression of GPC3 determined by immunohistochemistry in pancreatic ductal adenocarcinomas. This finding seemed unexpected to us since expression of GPC3 is something pathologists more commonly associate with hepatocellular carcinoma and yolk sac tumor as opposed to adenocarcinoma [4]. However, if GPC3 was indeed expressed in a significant proportion of pancreatic adenocarcinomas, it would have very significant implications beyond prognostication. Clinical trials are underway investigating GPC3 as a therapeutic target in hepatocellular carcinoma and a number of pediatric cancers [5]. This includes a recent trial using a chimeric antigen receptor-engineered T-cell (CAR-T)-based approach targeting GPC3 [6]. GPC3-targeted monoclonal antibodies (GC33 and 32A9) have been investigated in animal models for HCC and in early-phase clinical trials in human patients with HCC [7]. GPC3-derived peptide/DNA vaccines, gene therapy targeting GPC3, and GPC3-targeted human nanobody (HN3) immunotoxins are other therapeutic approaches under investigation for GPC3-expressing tumors [7]. While none of the GPC3-targeted therapies have shown a compelling therapeutic benefit in a phase 3 clinical trial, it seems to be a promising area of investigation. Combining one or more of these GPC3-based approaches with other immunotherapies such as checkpoint inhibitors is another possibility being explored [7].

Given the potential significance of the Yao study for GPC3 as a novel prognostic and predictive marker in pancreatic adenocarcinoma, we wanted to see if we could replicate their GPC3 results in pancreatic cancers in our Canadian patient population. In addition to GPC3, Yao et al. [2] tested tumors for CK19, which also had prognostic significance. We tested archived formalin-fixed paraffin-embedded pancreatic tissue in our laboratory archives for both markers and expanded the study population to include both pancreatic adenocarcinoma and neuroendocrine tumors (NETs). The primary objective of the study was to determine if we could independently identify GPC3 expression in pancreatic cancer in a significant proportion of patients in our Canadian patient population with the antibody protocol we currently use in diagnostic pathology practice.

## 2. Materials and Methods

### 2.1. Specimen Identification

This is a retrospective cohort study. Patients with a surgical resection for pancreatic adenocarcinoma or pancreatic NET between 1 January 2012 and 31 December 2016 were identified through an electronic search of anatomical pathology records (Cerner Millennium, Oracle Corporation, Kansas City, MI, USA) at the Queen Elizabeth II Health Sciences Centre, an academic tertiary care hospital in Halifax, NS, Canada. All consecutive patients during that time period who underwent surgical resection of the proximal, distal, or entire pancreas (Whipple pancreaticoduodenectomy, distal pancreatectomy, and total pancreatectomy) were included in the study cohort if they had a pathologic diagnosis of invasive adenocarcinoma or well-differentiated neuroendocrine tumor. The diagnoses included in the study cohort were pancreatic ductal adenocarcinoma not otherwise specified, ampullary adenocarcinoma, periampullary adenocarcinoma, intrapancreatic cholangiocarcinoma (adenocarcinoma), and well-differentiated neuroendocrine tumor. We did not include pancreatic core needle biopsies or biopsies of metastases in this study (due to limited tissue availability for research). We did not include resections for benign or in situ neoplasms, such as intraductal papillary mucinous neoplasm (IPMN) or mucinous cystic neoplasm, unless there was an invasive adenocarcinoma component. In cases with a precursor lesion such as IPMN, only the invasive adenocarcinoma component was part of the study tissue. Hematoxylin and eosin (H&E) slides for all cases were retrieved from the hospital pathology archives for review.

### 2.2. Case Review, Tissue Microarrays, and Immunohistochemistry

H&E slides from all cases were reviewed by a study pathologist (TA) with expertise in pancreaticobiliary pathology to confirm the diagnosis of adenocarcinoma or NET. The study pathologist identified and circled all tumors on H&E slides corresponding to archived formalin-fixed, paraffin wax-embedded tissue blocks. The tissue blocks were retrieved and duplicate 2 mm tissue cores were collected and placed in tissue microarrays using the Tissue-Tek Quick-Ray (Sakura Corporation, Bengaluru, India) microarray system. Immunohistochemical stains were performed on 4 μm thick sections cut from the tissue microarrays. Immunohistochemistry was applied using a Ventana Benchmark automated system (Ventana Medical System Inc., Tucson, AZ, USA) at the Queen Elizabeth II Pathology laboratory according to protocols previously validated for clinical diagnostic use of GPC3 and CK19. The Queen Elizabeth II Health Sciences Centre is a high-volume regional cancer center with centralization of surgical, pathology, and immunohistochemistry expertise in pancreaticobiliary cancers for the Atlantic Canadian region. The institution emphasizes centralization, sub-specialization, quality assurance, and multidisciplinary care, which are described as essential elements for a high-quality clinical cancer care program [8].

In brief, immunohistochemistry for GPC3 was performed using the Dako (Agilent Technologies, Inc., Santa Clara, CA, USA) A53-B/A2.26 antibody clone, at a 1:400 dilution. The Ultra Cell Conditioner 1 solution (Ventana) was applied for 36 min for antigen retrieval, and the iView DAB detection kit (Ventana) was used. Cases were defined as having positive expression when >10% of tumor cells showed at least weak-intensity staining as determined by the study pathologist reviewer using light microscopy. Human placental tissue and a hepatocellular carcinoma with known positive staining for GPC3 served as a same-slide positive control.

Immunohistochemistry for CK19 was performed using the Cell Marque (Sigma Aldrich, Inc., Rocklin, CA, USA) 1G12 antibody clone at a 1:75 dilution. The Ultra Cell Conditioner 1 solution (Ventana) was applied for 48 min for antigen retrieval and the OptiView detection kit (Ventana) was used. Cases were defined as having positive expression when >10% of cells showed at least weak-intensity staining as determined by the study pathologist reviewer. Bile ducts in human liver tissue with positive CK19 staining served as a same-slide positive control.

In the tumors that stained positive for CK19, we recorded both the proportion (percentage) of positive cells and the intensity of staining, based on a subjective scale of weak, moderate, or strong expression (strong being similar or stronger in intensity than CK19 in normal bile ducts).

Interpretation of immunohistochemistry for both CK19 and GPC3 was all carried out by a single pathologist reviewer (TA) who was blinded to demographic, staging, and outcome data for the study patients at the time of immunohistochemistry interpretation.

### 2.3. Clinical Information

Electronic medical records were reviewed to determine patient age at diagnosis, gender, and the surgical resection procedure for all patients. For the pancreatic NET cases, we retrospectively determined the TNM stage (including nodal and distant metastasis), tumor grade, and overall survival by review of electronic medical records, including pathology reports, radiology reports, and clinic notes. We did not review the electronic records for TNM stage, grade, or survival in the adenocarcinoma cohort since we did not have groups for comparison based on the CK19 or GPC3 expression result.

### 2.4. Statistical Analysis

Fisher’s exact tests (GraphPad Prism v.10.4.0, GraphPad Software, San Diego, CA, USA) were used to compare grade, stage, and overall survival in patients with NETs that were CK19-positive versus patients with NETs that were CK19-negative. Kaplan–Meier survival curves were generated and the log rank test was carried out using an online statistical calculator (astata.com; by Navendu Vasavada). For all statistical tests, *p* < 0.05 was considered statistically significant.

### 2.5. Ethics

The study protocol was approved by the research ethics board of the Nova Scotia Health Authority.

## 3. Results

Archived tissue from 72 patients with a pancreatic surgical resection at our institution with a diagnosis of adenocarcinoma and 20 patients with a pancreatic resection with a diagnosis of NET were identified. The 72 patients with adenocarcinoma included 32 (44%) females and 40 males (56%), with a mean age of 64.8 years at time of surgery (median 66 years, range 42–83 years). The seventy-two adenocarcinomas included fifty-six pancreatic ductal adenocarcinomas (not otherwise specified), eight ampullary adenocarcinomas, six periampullary adenocarcinomas, and five intrapancreatic cholangiocarcinomas. The surgical resections in this cohort included sixty-three Whipple pancreaticoduodenectomies, six distal pancreatectomies, and three total pancreatectomies. The 20 patients with well-differentiated neuroendocrine tumors included 5 females (25%) and 15 males (75%), with a mean age at diagnosis of 59.9 years (median 63 years, range 31–83 years). The surgical resections in the NET cohort included two Whipple pancreaticoduodenectomies and eighteen distal pancreatectomies. There were fourteen grade 1 NETs in the cohort and six grade 2 NETs. Archived formalin-fixed paraffin-embedded tissues from the surgical resections of all 72 adenocarcinomas and 20 NETs were tested with immunohistochemistry targeting GPC3 and CK19. The proportions of cases from each tumor type and the proportions with expression of GPC3 and CK19 are presented in Table 1.

**Figure 1:**
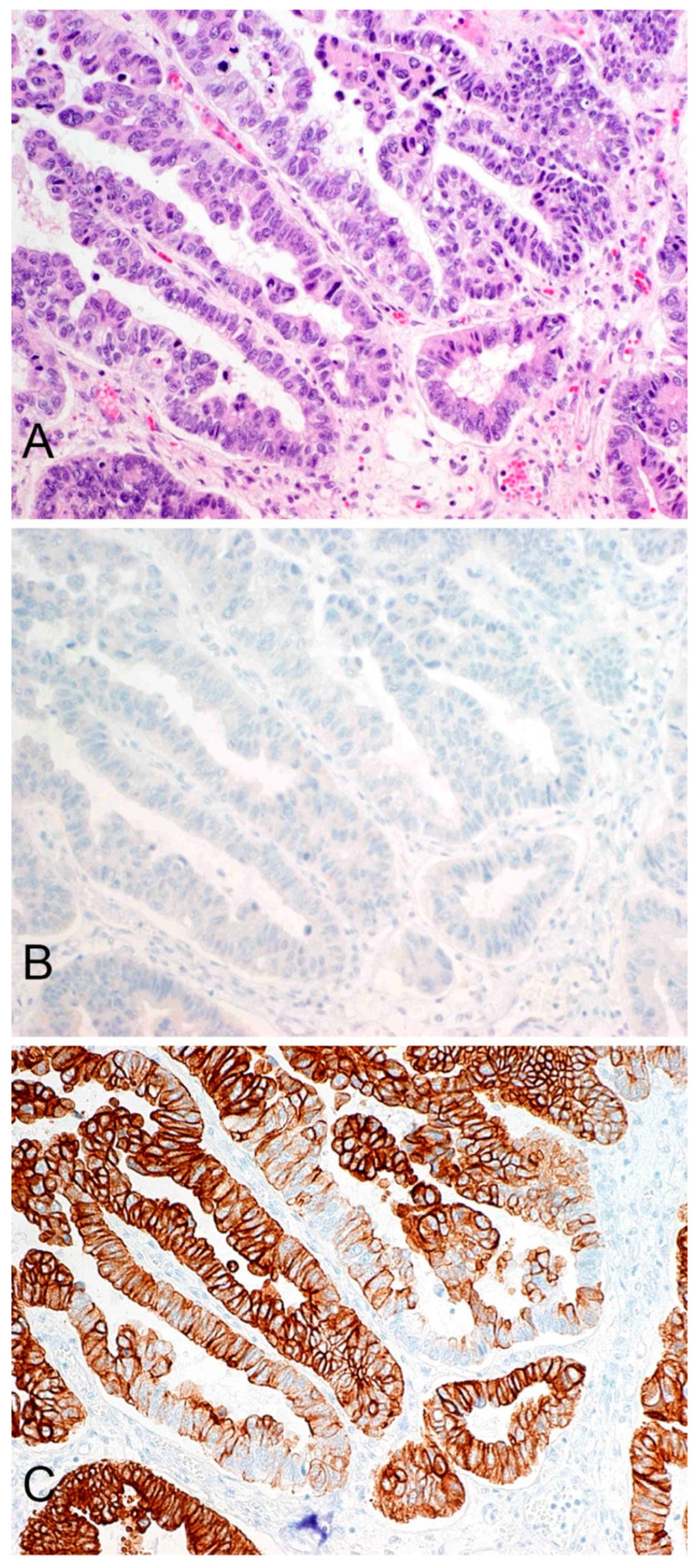
(**A**) H&E section of a representative case of pancreatic adenocarcinoma. (**B**) The tumor has no expression of GPC3, as determined by immunohistochemistry. (**C**) The tumor is strongly and diffusely positive for CK19, as determined by immunohistochemistry (all photos are original magnification× 200).

GPC3 expression was observed in 1/72 (1.4%) pancreatic adenocarcinomas and in 0/20 (0%) pancreatic NETs. The single positive adenocarcinoma had weak-intensity expression in 10% of tumor cells, a finding of uncertain significance. CK19 expression was observed in 72/72 (100%) pancreatic adenocarcinomas and 10/20 (50%) pancreatic NETs. Of the adenocarcinoma samples that tested positive for CK19 expression, 70/72 (97%) had strong-intensity staining and 2/72 (3%) had moderate-intensity staining. The proportion of cells staining positive ranged from 80 to 100%. Staining in a representative adenocarcinoma is illustrated in Figure 1. Of the pancreatic NET samples that tested positive for CK19 expression, 3/10 (30%) had strong-intensity expression, 6/10 (60%) had moderate-intensity expression, and 1/10 (10%) had weak-intensity expression. The proportion of tumor cells staining positive ranged from 10–100%.

Since almost no tumors expressed GPC3, and all adenocarcinomas expressed CK19, we did not carry out clinical follow-up or a statistical analysis to correlate the expression of these markers with stage, grade, or overall survival. In the NETs, we compared stage, grade, and overall survival in the group of patients with CK19-expressing tumors compared to the group with CK19-negative tumors. The relevant clinicopathological information is presented in Table 2.

**Figure 2:**
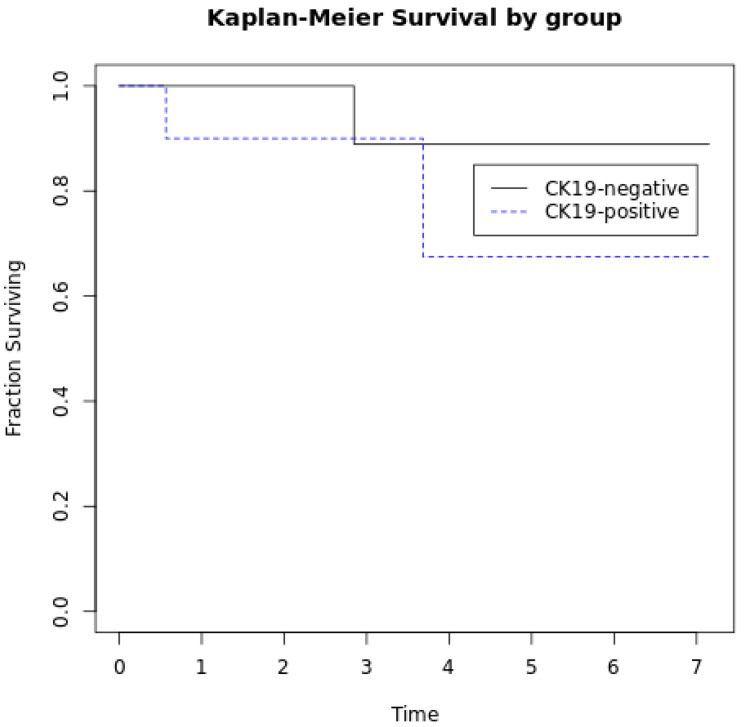
Kaplan–Meier overall survival curves comparing survival in the cohort of patients with CK19-negative NETs and CK19-positive NETs. There was no difference in overall survival between the two cohorts (*p* = 0.41, log rank test). Time on the *x*-axis is in years.

There was no statistically significant correlation between CK19 expression with the presence or absence of lymph node metastasis (*p* = 0.63), distant metastasis, and/or lymph node metastasis (*p* = 0.35), or higher grade (*p* = 0.18) in the NET cohort. The mean follow-up time for the patients in the NET cohort was 3.7 years. Two of the ten patients in the CK19-positive group died during follow-up and one of the ten patients in the CK19-negative group died. There was no difference in the proportion of patients surviving in the two groups (*p* = 1.00, Fisher’s exact test). There was also no difference in Kaplan–Meier overall survival curves of the CK19-negative and CK19-positive cohorts based on the log rank test (*p* = 0.41). The Kaplan-Meier survival curves are shown in Figure 2.

## 4. Discussion

The purpose of this study was to determine the proportion of pancreatic adenocarcinomas and pancreatic NETs with GPC3 and CK19 expression in our Canadian population and to determine if we could find prognostic value in these markers, as was previously reported by Yao et al. [2]. In the adenocarcinomas tested by Yao et al., GPC3 expression was observed in 47 of 106 (44%) pancreatic adenocarcinoma tumor samples tested, and CK19 expression was observed in 63 of 106 (59%) pancreatic adenocarcinomas. They found that the expression of GPC3 predicted larger tumor size, more advanced TNM staging, and increased lymph node involvement. They found that the positive expression of CK19 was associated with increased lymph node involvement and poorer tumor differentiation [2]. Since all of the pancreatic adenocarcinoma cancer samples we tested in our population had positive expression for CK19 and negligible expression of GPC3 (only a single case had weak expression of GPC3 in 10% of cells), we believe that these stains are not useful prognostic markers in our population when using our clinical protocols for these stains. More importantly, we think that our results suggest that pancreatic cancer in our population is a less promising target for GPC3-targeted immunotherapies than previously thought.

It is difficult to know why there are such large differences in the results of our study and those of Yao et al. There are differences in the immunohistochemical staining protocol that may be contributory. Our study’s protocol used a Ventana detection kit, while Yao et al. used a Dako (Carpintiera, CA, USA) detection kit [2]. Perhaps more importantly, the primary antibody clones used for CK19 and GPC3 might be different. Unfortunately, the specific antibodies used are not stated in the Yao protocol. We used the A53-B/A2.26 and 1G12 clones that are validated for diagnostic pathology practice at our hospital. Non-specific staining is also a possibility in the Yao study. For example, they note GPC3 staining in some areas of chronic pancreatitis and benign peritumoral tissues, suggesting that their staining protocol may be reacting non-specifically.

While there are no studies other than Yao’s study and our study that attempted to look at GPC3 expression as a potential prognostic and predictive marker for pancreatic adenocarcinoma, there are some studies where a rate of expression of GPC3 in pancreatic adenocarcinoma can be found. Typically, these are studies looking at the value of GPC3 as a site-specific marker to determine if a tumor is of hepatocellular origin or if it could represent a metastatic carcinoma from another site. In a study by Moek et al., 0 of 89 (0%) pancreatic adenocarcinoma samples tested had GPC3 expression [4]. A study by Mounajjed et al. reported that 0 of 26 (0%) pancreatic NETs and 0 of 22 (0%) pancreatic adenocarcinomas had GPC3 expression. Their study also found that, in general, the only pancreatic tumor that expresses GPC3 is acinar cell carcinoma [3].

Interestingly, a related glypican protein known as glypican-1 (GPC1) has recently shown promise in the literature as a biomarker in pancreatic cancer. Several studies have demonstrated that GPC1 is overexpressed in pancreatic adenocarcinoma [9]. The expression of GPC1 in pancreatic tumors and serum extracellular vesicles has also been found to be associated with poorer prognosis and higher stage and grade disease [10]. GPC1 may ultimately prove to be a more fruitful area for future pancreatic cancer research than GPC3.

There are prior studies of CK19 expression in pancreatic adenocarcinoma that report similar data to our findings. For example, in a study by Zapata et al., 23 of 25 (92%) of the pancreatic adenocarcinoma samples tested showed positive expression for CK19 [11]. In addition, according to a review article by Jain et al., most pancreatic and gastrointestinal adenocarcinomas display positive CK19 expression [12]. This supports our view that CK19 is so commonly expressed in pancreatic adenocarcinomas that it seems very unlikely to provide any useful prognostic information in clinical practice.

In our study, 10 of 20 (50%) of the pancreatic NETs tested had CK19 expression. There are prior studies that demonstrate a proportion of pancreatic NETs are CK19-positive. For example, in a study by Son et al., CK19 expression was recorded in 97 of 182 (53.3%) pancreatic NETs tested [13]. In a study by Han et al., 70 of 100 (70%) pancreatic NETs tested had positive CK19 expression [14]. In another study by Salla et al., 25 of 28 (89.3%) pancreatic NETs tested positive for CK19 expression [15]. Other studies have shown CK19 expression within ranges of 49–70% in NETs [13]. There is some evidence in the literature suggesting that CK19 expression may be an adverse prognostic marker in pancreatic NETs [13]. In the study by Han et al., CK19 expression was associated with worse overall survival [14]. This differs from the findings of the study by Son et al., which found no significant survival difference in patients who had tumors expressing CK19 and those who did not. Both of these studies, however, found an association between positive CK19 expression and increased regional lymph node metastases, lymphovascular invasion, and higher TNM staging [13]. We did not find CK19 to have prognostic value in the population of patients with pancreatic NETs that we tested. However, our study is significantly limited by the amount of follow-up data we have available as well as the limited sample size of the population we tested. Our study is not powered to adequately assess CK19 as a prognostic marker in pancreatic NETs.

## 5. Conclusions

The very low rate of GPC3 expression we observed suggests that patients with pancreatic cancer in our population would be unlikely to benefit from novel GPC-targeted therapeutics. Admittedly, there is a possibility that different antibody clones in different patient populations might reach different conclusions. As a prognostic marker, CK19 might have the most potential in the population of patients with NETs given its variable expression in these tumors and the results of previous studies. CK19 expression did not show prognostic significance in our population, but that may be due to our limited sample size and follow-up. Larger studies of this marker may reach different conclusions.

## Figures and Tables

**Table 1:** Number of adenocarcinomas and NETs which were positive for GPC3 and CK19, as determined by immunohistochemistry, number of tumors tested, and proportion (%) of cases that were positive.

Tumor Type	GPC3			CK19		
	Number+	Number Tested	%	Number+	Number Tested	%
NET	0	20	0	10	20	50
Adenocarcinoma	1	72	1.4	72	72	100

**Table 2:** Clinicopathological characteristics of NET cases.

Clinical Data	Case Number (*n* = 20)	Percentage (%)
Age		
<50	5	25
50–64	9	45
65+	6	30
Gender		
Male	15	75
Female	5	25
pT Status		
T1	8	40
T2	9	45
T3	3	15
pN Status		
NX	3	15
N0	13	65
N1	4	20
pM Status		
MX	3	15
M0	15	75
M1	2	10
Tumor Grade		
1	14	70
2	6	30
3	0	0
Survival *		
Deceased	3	15
Living	17	85

* Survival status at last available follow-up.

## Data Availability

The original contributions presented in the study are included in the article, further inquiries can be directed to the corresponding authors.
